# Reliability and validity evaluation of the Chinese version of the gender misconceptions of men in nursing (GEMINI) scale among nursing students

**DOI:** 10.1186/s12912-024-01939-4

**Published:** 2024-04-24

**Authors:** Huameng Xu, Chunguang Liang, Jie Kong, Qing Chen, Ying Zhao, Fan Zhang

**Affiliations:** School of Nursing, Jinzhou Medical University, No 40, Section 3, Songpo Road, 121001 Jinzhou, China

**Keywords:** Gender misconception, Gender bias, Scale, Instrument, Nursing student, Men in nursing

## Abstract

**Background:**

Misconceptions about male nurses not only exacerbate the gender imbalance in the nursing profession but also negatively impact male nurses embarking on their careers. Currently, no tool exists to measure the gender biases toward males in nursing among nursing students in China. Consequently, the primary objective of this study is to assess the validity and reliability of the Chinese translation of the Gender Misconceptions of Men in Nursing (GEMINI) scale among nursing students.

**Methods:**

This cross-sectional study involved 1,102 nursing students from China who participated online. We utilized the Brislin translation technique with a forward-backward approach. To determine the factor structure within the Men in Nursing Gender Misconceptions Scale’s Chinese version, both exploratory (EFA) and confirmatory factor analysis (CFA) were applied. The scale’s internal consistency was measured through the Cronbach’s alpha coefficient, corrected item-total correlation, and a retest reliability assessment.

**Results:**

The scale showed a content validity index of 0.938 and a retest reliability of 0.844. EFA indicated a two-factor structure for the translated instrument. CFA revealed a chi-square/degree of freedom of 3.837, an incremental fit index (IFI) of 0.952, a goodness-of-fit index (GFI) of 0.910, a comparative fit index (CFI) of 0.952, and an RMSEA of 0.073, all of which were within acceptable limits. The scale’s Cronbach’s *α* was 0.953, and the corrected item-total correlations ranged between 0.539 and 0.838. Gender-based misconceptions about men in nursing among students appeared to be influenced by their gender and whether they considered a nursing program as their first choice when applying for a major. Misconceptions about male nurses are greater among men and those who do not consider nursing programs as their first choice when applying for a major.

**Conclusions:**

The Chinese adaptation of the GEMINI scale showcased high reliability and validity. It stands as a potential instrument to gauge gender misconceptions concerning male nurses among Chinese nursing students.

## Background

With the development of social medicine and health care, the demand for medical and nursing resources in medical units continues to show a diversified trend [1]. Although the number of nurses is increasing, the structure of the nursing team remains suboptimal. The nursing profession requires not only female nurses but also a substantial number of male nurses. Despite some progress in achieving gender diversity, men are still underrepresented in nursing [2]. Internationally, the proportion of male nurses rarely exceeds 10% [3, 4], with figures ranging from 11% in the UK (2017) [5, 6] and 7% in Canada (2016) [5] to 9.6% in the US (2018), 10.5% in Sweden [7], 11.7% in Australia (2018) [8], and 16.7% in Spain [8, 9]. In China, the proportion of male nurses has been increasing annually; however, a significant gender gap persists. In 2020, male nurses constituted only 3.4% of the nursing workforce, while female nurses accounted for 96.6% [10]. Given the persistent underrepresentation of men in the nursing workforce, an increase in male nurses is necessary to address the growing gender imbalance in the profession [11].

Since the era of Florence Nightingale in the nineteenth century, nursing has historically been viewed as a female-dominated field [12]. According to Florence Nightingale, nursing was the most suitable profession for women [13]. She posited that providing care was an extension of motherhood and considered women more adept at it. As a result, male individuals aspiring to become nurses often face challenges to their perceptions of masculinity from others [14, 15]. This is manifested through a lack of understanding, being the subject of conversations, or even verbal abuse, such as being labeled perverted or effeminate.

Although society promotes gender equality, there remains a widespread gender imbalance in certain professions due to historical gender biases. Men are consistently under-represented in the nursing, teaching, and social work professions, particularly in nursing [16]. Even in recent years, the percentage of male nurses has increased and has become more socially acceptable in terms of treatment and development [17]. However, the nursing workforce still faces significant challenges due to deeply ingrained prejudices and misunderstandings about males working in the nursing field [18]. These misconceptions have led to many difficulties and distress for male nurses in clinical work, such as rejection and refusal by patients and female colleagues, resulting in increased turnover, reduced job satisfaction for male nurses, and exacerbation of the nursing gender imbalance [19].

In society, men are perceived as highly expressive and assertive, while women are seen as more hospitable and kind [16]. Most individuals tend to pursue careers that align with their interests and skills. Many men choose fields such as physics, computer science, and engineering, while women prefer careers in nursing, teaching, and social work [20, 21]. Nursing is not always the first career choice for male nursing students, some may only consider it after not succeeding in other programs [1]. Male nursing students are often mistaken for medical students or doctors [22], potentially because patients are unaware that men can also be nurses [23]. Furthermore, some male nurses feel isolated from their student days through to their clinical work [24]. Additionally, male nursing students frequently encounter female patients who refuse their services during clinical placements, especially in obstetrics and gynecology [25]. These studies indicate that male nurses tend to be assigned to technical, low-touch, and administrative roles. This dynamic deters potential male students from choosing nursing as a career.

To address the shortage of male nurses in the workforce and to achieve gender diversity in nursing, it is essential to examine the female-dominated image of nursing, both in the clinical setting and as perceived by the general public. Changing the perception of nursing as feminine and eliminating professional gender stereotypes and misconceptions about men will enable male nurses to reach their full professional potential. There is a lack of tools to assess gender misconceptions among male nurses. Identifying appropriate tools for evaluating misconceptions about men in nursing could help address the critical issue of the shortage of male nurses in the global workforce.

The questionnaires related to the gender of nurses in China currently include the Gender Stereotypes of the Nursing Profession Questionnaire, but this tool does not comprehensively summarize the relevant information about gender misconceptions of nurses and can only be examined from the perspective of stereotypes. Given that gender misperceptions of male nurses also exist in China without a relevant tool, there is an urgent need for a tool that can assess gender misperceptions of male nurses.

The Gender Misconceptions of Men in Nursing Scale (GEMINI), originally developed by Jed Montayre et al. in Australia, provides a quick and efficient method for nursing students to assess their misconceptions about males in nursing [26]. The GEMINI scale evaluates and identifies factors that contribute to misunderstandings to support the retention of these students and reduce the attrition rate. The GEMINI has demonstrated good reliability, validity, and accuracy in Australia. In this study [26], participants were recruited from 16 nursing institutions, showing that men, especially those who did not choose nursing as their primary career choice, were in their final year of school, and worked for pay in the healthcare industry, exhibited more gender misconceptions.

The primary objective of this study was to translate the GEMINI into Chinese and subsequently evaluate its reliability and validity among Chinese nursing students. By doing so, we aimed to assist Chinese nursing education in bridging the gender gap prevalent in nursing professions, thereby diversifying career pathways. This could potentially increase the number of male nurses, addressing the shortage of male nurses in the workforce. Furthermore, we postulated that the perceptions or misperceptions of Chinese nursing students regarding men in the nursing field would relate to specific cultural and social environmental characteristics in China. To corroborate this, we analyzed the variance in data sets used to compute scores for the Chinese version of the GEMINI and highlighted notable discrepancies among them.

## Methods

### Study design and participants

This cross-sectional study was conducted between January and March 2023, using a convenient sample of nursing students. Some participants were studying nursing in medical schools, while others were enrolled in comprehensive universities. The majority of these students were from Shenyang Medical College, Jinzhou Medical University, China Medical University, Dalian Medical University, and were nursing students completing their internships in Liaoning Province. These universities are located in Liaoning Province. Data were collected through the online data collection platform, Questionnaire Star, in China. Test-retest reliability was assessed. The researcher analyzed the data and excluded 93 questionnaires that were clearly logically inaccurate and did not meet the study requirements. Ultimately, a total of 1,102 questionnaires were completed. The survey was anonymous; however, to assess test-retest reliability after two weeks, thirty participants were asked to record their contact information.

The GEMINI consists of 17 items. Since a total of 1,532 subjects were collected for the original scale, the sample size was increased to ensure a reduction in error from the original scale. All participants are native Chinese speakers. The participants provided informed consent before their involvement in the study. Both the Jinzhou Medical University Ethics Committee’s (Grant Number: JZMULL2021009) and the 1964 Helsinki Declaration and its later amendments were adhered to in the research methods [27].

### Instruments

#### Professional identity questionnaire for nurse students

The Professional Identity Questionnaire for Nurse Students (PIQNS) was developed by Yufang Hao with 17 entries and 5 dimensions, namely self-concept, benefit of staying and risk of leaving, social support and self-reflection, autonomy of career choice, and social persuasion. The Likert-5 scale was used, with higher scores indicating a stronger career identity of nursing students. The Cronbach’s *α* for the questionnaire in their article was 0.827. The questionnaire has demonstrated good reliability and validity [28].

#### The gender stereotypes of the nursing profession questionnaire

The Gender Stereotypes of the Nursing Profession Questionnaire was developed by Bi-Hui He et al. and consists of 2 entries [29]. Item 1 assesses the perceived suitability of men for the nursing profession, and item 2 evaluates the perceived importance of gender to the nursing profession. The items were scored on a scale of 1 to 3, with the total score of the scale being the sum of the scores of each item. The higher the score, the deeper the stereotype of the nursing profession. The scale scores range from 2 to 6, with scores of 2 to 3 indicating a low level of stereotypes, 4 indicating a medium level, and 5 to 6 indicating a high level. The scale has demonstrated good content validity, and the retest reliability is 0.85, indicating good reliability.

#### Demographic characteristic

All participants completed three questionnaires: the Chinese version of the Gender Misconceptions of Men in Nursing (GEMINI) scale, the Gender Stereotypes of the Nursing Profession Questionnaire, and the Professional Identity Questionnaire for Nurse Students (PIQNS). Additionally, participants provided relevant demographic information, including gender, age, education, school year, and place of residence.

### Procedures

#### Translation procedure

Initially, two Chinese Master’s students specializing in English translated the GEMINI scale into Chinese, prioritizing cultural relevance. This translation process commenced after obtaining consent from the original scale’s author and followed the Brislin method of forward-backward translation [30]. A psychologist and four nursing specialists from the Liaoning area, each with over a decade of experience, subsequently reviewed the original questionnaire, its initial Chinese version, and the re-translated English variant. These experts, proficient in both Chinese and Western cultures, aimed to refine the questionnaire to better reflect Chinese cultural norms. In the final validation phase, 10 students from Jinzhou Medical University, chosen through convenience sampling, provided feedback on the revised scale, leading to further improvements based on their insights.

#### Data collection procedure

Participants accessed and completed several online questionnaires via provided links, including the GEMINI scale, the Nursing Professional Gender Stereotyping Questionnaire, the Professional Identity Questionnaire for Nursing Students (PIQNS), and sociodemographic data sheets.

### Statistical analysis

SPSS 25.0 and AMOS 26.0 (IBM Corporation) were employed for data analysis. Differences in the Chinese versions of the GEMINI scores across sociodemographic and clinical variables were assessed using independent samples t-tests or one-way ANOVA, with Bonferroni tests for significance correction in pairwise comparisons. A significance threshold of *P* < 0.05 was maintained. The skewness and kurtosis for each item were computed, and data were considered normally distributed if values ranged from − 2 to + 2 [31].

### Validity analysis

#### Content validity

Content validity evaluates how well the actual content measured aligns with the intended content [32]. Experts used a 4-point scale (1 = irrelevant, 2 = weakly relevant, 3 = strongly relevant, 4 = very strongly relevant) to determine the relevance of each item to its respective dimension, facilitating the calculation of the CVI.

#### Structure validity

The construct validity of the Chinese version of the GEMINI scale was assessed using EFA and CFA. The total sample of 1102 cases was randomly divided into two groups: 539 participants completed the EFA, and 563 completed the CFA.

In the first group (*n* = 539), the internal structure of the translated Chinese GEMINI version was evaluated using Principal Component Analysis (PCA) with varimax rotation. The Kaiser-Meyer-Olkin (KMO) [33] measure and Bartlett’s test of sphericity [34] were used to assess the sample’s adequacy for factor analysis. A KMO value greater than 0.6 (*P* < 0.05) and a significant Bartlett’s test indicated that the sample was suitable for analysis. Factors with eigenvalues greater than 1 underwent maximum variance orthogonal rotation. Items with loadings of 0.40 or higher were considered for inclusion in separate factors [35]. Factor extraction was guided by eigenvalues, the total variance explained, and the scree plot.

In the second group (*n* = 563), CFA was utilized to validate the results of the EFA or to further assess the measurement model. CFA aids in evaluating the model’s fit based on the established factor structure [36]. The model’s goodness of fit was assessed using several indices: Chi-square (χ2) to degrees of freedom (df), root mean square error of approximation (RMSEA), standardized root mean square residual (SRMR), normed fit index (NFI), goodness of fit index (GFI), and comparative fit index (CFI) [37]. An acceptable model fit was defined by a χ2/df < 3, RMSEA and SRMR < 0.08 [38], and GFI, CFI, and an IFI > 0.90 [39].

#### Discriminant validity

Discriminant validity evaluates the distinctiveness of different latent variables [40]. For the GEMINI scale, the total scores were ranked in ascending order. The lowest 27% of scores constituted the low score group, and the highest 27% made up the high score group. Differences in item scores between these groups were analyzed using a two-tailed independent samples t-test.

### Reliability analysis

#### Internal consistency reliability

The scale’s internal consistency was assessed using several methods, including the Cronbach’s alpha coefficient, corrected item-total correlation, and retest reliability. A Cronbach’s alpha value of 0.70 or higher is generally deemed acceptable [41]. A threshold of 0.3 was set for the corrected item-total correlation, representing each item’s correlation with the sum of the remaining items on the scale [42]. Retest reliability was evaluated using the intraclass correlation coefficient (ICC), which measures the scale’s stability over time.

#### Test-retest reliability

To assess test-retest reliability, 30 adults who had previously taken the test two weeks earlier were recruited. The correlation between their initial and subsequent test scores was evaluated using Spearman’s correlation. A correlation coefficient of 0.7 or higher was set as the cutoff point [43].

## Results

### Descriptive statistics

The study involved 1,102 nursing students, predominantly females (89.2%) and first-year students (56.4%). Nursing was the first choice for 73.2% of participants, and 68.1% were not engaged in paid work. Additional demographic details are provided in Table 1. Table 2 displays the mean (SD) scores for each item on the Chinese version of the GEMINI. The skewness and kurtosis values suggested that the data were normally distributed.

**Table 1:** Demographic characteristics of participants (*N* = 1,102)

Characteristics	N	%
Sex		
Male	119	10.8
Female	983	89.2
Age(mean, standard deviation)	21.6 ± 2.3	
School Year		
First year of specialization	234	21.2
Second year of specialization	4	0.4
Third year of specialization	13	1.2
1st grade	622	56.4
2nd grade	99	9.0
3rd grade	49	4.4
4th grade	14	1.3
First year graduate students	3	0.3
Second year graduate students	9	0.8
Third year graduate students	3	0.3
Others	52	4.7
Academic qualifications		
College	251	22.8
Undergraduate	816	74.0
Master or above	16	1.5
Others	19	1.7
Reasons for enrolling in the nursing program		
Voluntary	498	45.2
Parental wishes	218	19.8
forced by reality	198	18.0
Others	188	17.1
Is nursing your first choice		
Yes	807	73.2
No	295	26.8
Immediate family member in the nursing profession		
Yes	178	16.2
No	924	83.8
Whether there is pressure to learn while studying nursing		
Yes	729	66.2
No	373	33.8
Nursing programme as first choice		
Yes, nursing programme was my first choice	882	80.0
No, another programme of study was first choice	220	20.0
Paid work engagement and type		
No, not engaging in any paid work during term-time	750	68.1
Yes, health-related employment during term-time	272	24.7
Yes, non-health-related employment during term-time	80	7.3

**Table 2:** Mean (SD) scores with skewness and kurtosis figures for the GEMINI scale (*N* = 1,102)

Item	Mean(SD)	Skewness	Kurtosis
1	2.43(1.233)	0.470	-0.565
2	2.74(1.175)	0.149	-0.513
3	2.11(1.129)	0.763	-0.080
4	2.12(1.107)	0.721	-0.094
5	1.82(1.040)	1.073	0.479
6	1.97(1.079)	0.876	0.102
7	2.65(1.211)	0.216	-0.654
8	2.59(1.254)	0.320	-0.721
9	2.39(1.171)	0.418	-0.529
10	2.47(1.157)	0.333	-0.455
11	2.22(1.092)	0.522	-0.299
12	2.17(1.125)	0.648	-0.258
13	2.01(1.036)	0.738	0
14	2.41(1.120)	0.344	-0.428
15	2.26(1.067)	0.476	-0.244
16	2.18(1.072)	0.545	-0.250
17	2.37(1.109)	0.332	-0.470

### Validity analysis

#### Content validity

Content validity for the Chinese version of the GEMINI was assessed by a panel of seven experts [44]. The results showed an I-CVI range from 0.813 to 1.000 and an S-CVI/Ave of 0.938.

#### Exploratory factor analysis

**Fig. 1:**
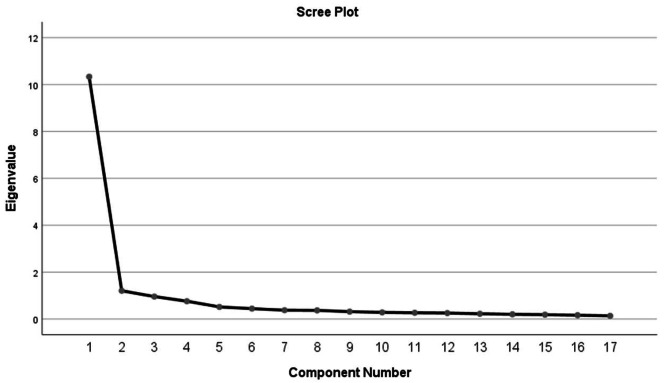
Scree plot of exploratory factor analysis for Chinese version of the GEMINI

The Bartlett’s test [34] of sphericity yielded a significant outcome (*P* < 0.001), and the KMO index [33] surpassed the minimum threshold of 0.6, confirming the suitability of the dataset for factor analysis. Principal component analysis with varimax rotation identified two common factors that explained 67.883% of the variance. This finding deviated from the original scale’s one-factor structure, with all 17 items loading across two factors (loadings ranging from 0.539 to 0.874). The results are detailed in Table 3, and the scree plot, shown in Fig. 1, further supported the two-factor structure by indicating a diminishing trend after the second point.

**Table 3:** Exploratory factor analysis (*N* = 539)

Original structure	Factor1	
Modified structure	Factor1	Factor2
13. Men nurses are often ostracised (isolated) by female nurses in the clinical settings	0.874	
6. Men have less opportunities for advancement in nursing than women	0.823	
16. As a minority group, it is difficult for men to be successful in nursing	0.789	
5. Men who choose nursing as a career are mostly gay	0.779	
12. Men in nursing are often just used as “muscles” by their female nurses	0.763	
15. Men nurses often experience communication difficulties with other healthcare professionals	0.746	
11. Men who are nurses are not taken seriously by other health professionals	0.713	
4. Nursing erodes the masculine identity of men	0.687	
17. Nursing is not an appropriate profession for men from certain cultural and religious groups	0.652	
3. Nursing is often a “dead-end” job for men	0.652	
14. Patients are generally reluctant to be nursed by men nurses	0.641	
8. I would not encourage a male family member (e.g. brother, son or cousin)to choose nursing as a career		0.824
7. The mass media (e.g. television and movies) puts most men off nursing		0.753
9. Compared to other health professionals (e.g. physiotherapist, dietitian, podiatrist), nursing is a low status job for men		0.703
10. Men should choose other professions that pay more than nursing		0.693
2. Being caring does not come naturally for men in nursing		0.690
1. Men are less suited to nursing as a career than women		0.539

#### Confirmatory factor analysis

**Fig. 2:**
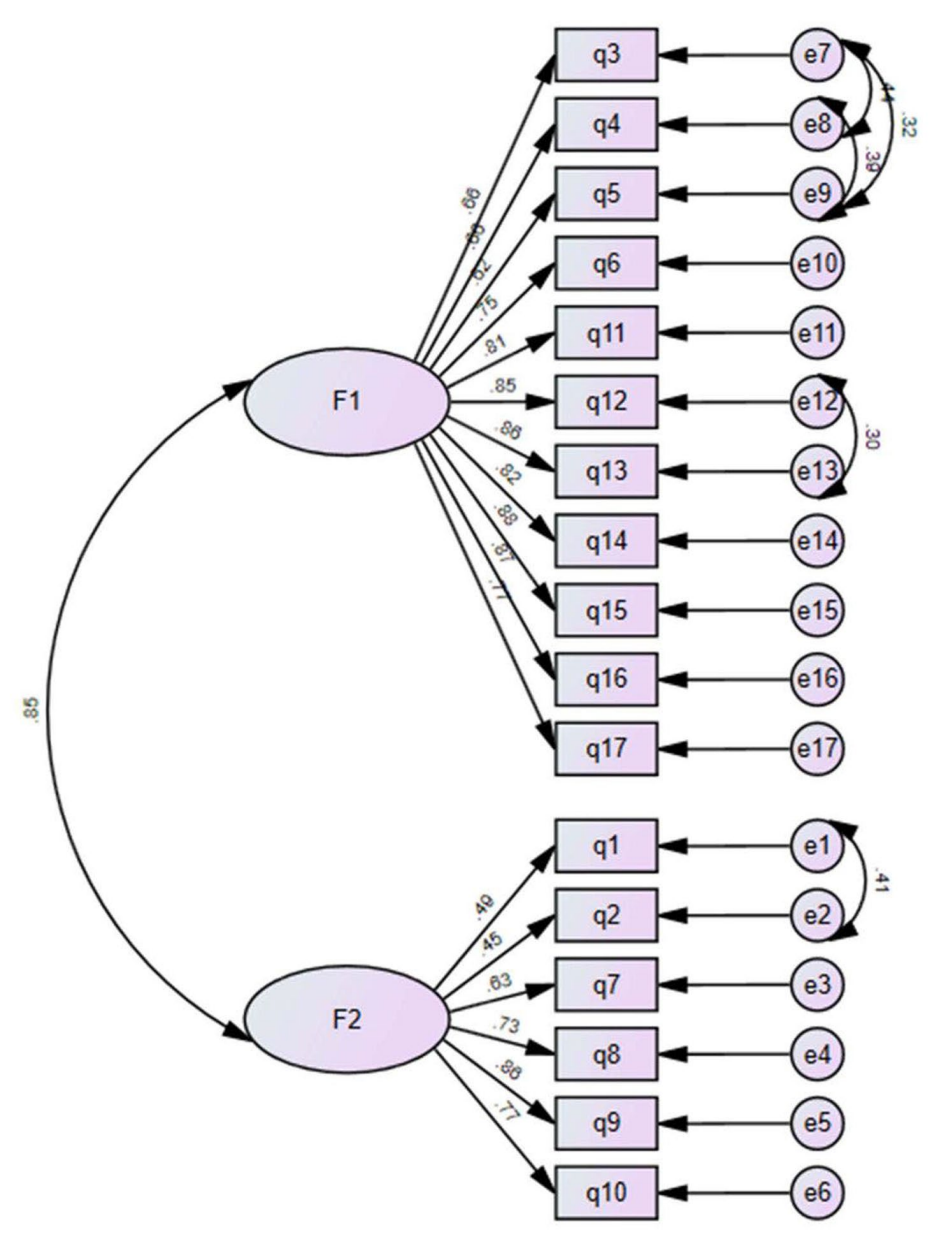
Standardized two-factor structural model of the GEMINI (*n* = 563). F1 (self development, eleven items), F2 (social acceptance, six items)

Confirmatory factor analysis was conducted on 1,102 nursing students using a two-factor model, which demonstrated a good fit to the data. Fit indices included: χ2/df = 3.837, CFI = 0.952, GFI = 0.910, AGFI = 0.879, PGFI = 0.672, IFI = 0.952, TLI = 0.942, RMSEA = 0.073, and RMR = 0.056. Figure 2 illustrates the results of the CFA.

#### Discriminant validity

Discriminant validity was evaluated using the extreme grouping method, which divided respondents into high and low-scoring groups (top 27% and bottom 27%). Threshold scores of 29 and 51 distinguished these groups, and mean item scores for each group were calculated. Two-tailed independent samples t-tests indicated significant differences between the groups for all items (*p* < 0.05). The specific statistical results are listed in Table 4.

**Table 4:** Score comparison between high-score and low-score groups (*N* = 1,102)

Item	Low-score group (*n* = 301), Mean (SD)	High-score group (*n* = 311), Mean (SD)	t-test(df)	*p*-value
1	1.50 (1.076)	3.26 (0.911)	-21.749 (587.250)	< 0.001
2	1.86 (1.234)	3.32 (0.822)	-17.182 (520.170)	< 0.001
3	1.13 (0.476)	3.27 (0.841)	-38.904 (493.237)	< 0.001
4	1.18 (0.608)	3.24 (0.835)	-34.873 (566.788)	< 0.001
5	1.07 (0.449)	2.92 (0.980)	-30.069 (437.565)	< 0.001
6	1.04 (0.308)	3.14(0.884)	‘-39.333 (386.284)	< 0.001
7	1.60 (1.096)	3.39 (0.803)	-22.999 (549.258)	< 0.001
8	1.41 (0.933)	3.46 (0.837)	-28.564 (610)	< 0.001
9	1.16 (0.555)	3.40 (0.776)	-41.219 (561.889)	< 0.001
10	1.32 (0.778)	3.41 (0.785)	-33.030 (609.665)	< 0.001
11	1.11 (0.477)	3.26 (0.799)	-40.529 (508.807)	< 0.001
12	1.05 (0.254)	3.35 (0.808)	-47.822 (372.471)	< 0.001
13	1.04 (0.249)	3.14 (0.804)	-43.911 (370.736)	< 0.001
14	1.26 (0.674)	3.38 (0.813)	-35.058 (595.977)	< 0.001
15	1.13 (0.442)	3.28 (0.755)	-43.090 (503.446)	< 0.001
16	1.12 (0.443)	3.23 (0.780)	-41.334 (494.323)	< 0.001
17	1.24 (0.679)	3.26 (0.757)	-34.813 (606.557)	< 0.001

#### Correlations among factors

Correlation analysis (Table 5) revealed a positive correlation between the total score and each dimension of the Chinese version of the GEMINI, as well as a negative correlation between the dimension scores, the total score, and the PIQNS scores.

**Table 5:** Pearson’s correlations between the Chinese version of GEMINI and PIQNS

	F1	F2
GEMINI	0.972**	0.906**
PIQNS	− 0.160**	− 0.202**

* Correlation is significant at the 0.05 level (2-tailed).

** Correlation is significant at the 0.01 level (2-tailed).

### Reliability analysis

#### Internal consistency reliability

Reliability analysis revealed that the Chinese translation of the GEMINI exhibited excellent internal consistency, with an overall Cronbach’s alpha coefficient of 0.952. The Cronbach’s alpha coefficients for the two factors were 0.852 and 0.951 respectively. These coefficients exceeded the minimum acceptable value [41]. Table 6 presents the correlation coefficients between the 17 questionnaire items and total scores, along with Cronbach’s alpha coefficients after removing individual items—all of which remained lower than the original Cronbach’s alpha coefficient of 0.952. Furthermore, corrected item-total correlations for all items exceeded 0.3 [42], leading to the retention of all 17 items without deletion.

**Table 6:** Cronbach’s alpha coefficient if the item was deleted and corrected item-total correlation (*N* = 1,102)

Item	Cronbach alpha if the item was deleted	Corrected item-total correlation
1	0.952	0.572
2	0.953	0.510
3	0.949	0.754
4	0.949	0.740
5	0.950	0.665
6	0.949	0.737
7	0.952	0.595
8	0.951	0.660
9	0.948	0.774
10	0.949	0.728
11	0.948	0.804
12	0.947	0.826
13	0.948	0.803
14	0.949	0.759
15	0.948	0.807
16	0.948	0.792
17	0.949	0.725

#### Test-retest reliability

After a two-week interval, the Chinese iteration of the GEMINI scale demonstrated robust test-retest reliability, evidenced by a Spearman correlation coefficient of 0.844, which exceeded the 0.7 threshold. This assessment involved 30 randomly selected adults who had previously participated in the survey and were asked to complete the questionnaire again.

### Analysis of differences in the Chinese Version of GEMINI based on sociodemographic characteristics

Significant differences in scores on the Chinese GEMINI version were observed with regard to certain demographic variables. Notably, differences were found based on gender and whether nursing was chosen as the primary major preference during the application process. Scores on the GEMINI were higher among men and among those for whom the nursing program was not their first choice. Conversely, no statistically significant differences emerged in the total score with respect to the other demographic variables in relation to the Chinese GEMINI version. A comprehensive summary of these findings is presented in Table 7.

**Table 7:** Comparison of the Chinese version of the GEMINI of subjects with different characteristics

Variable		Mean (SD)	t/F	*p*-value
Gender	Male	46.64 (16.012)	6.275	< 0.001
	Female	37.98 (13.986)		
Is nursing your first choice	Yes	38.57 (14.752)	-1.295	0.196
	No	39.85 (13.621)		
Immediate family member in the nursing profession	Yes	38.39 (16.802)	-0.464	0.643
	No	39.02 (13.976)		
Whether there is pressure to learn while studying nursing	Yes	39.29 (14.612)	1.203	0.229
	No	38.18 (14.157)		
Nursing programme as first choice	Yes	38.29 (14.564)	-2.879	0.004
	No	41.42 (13.801)		
Paid work engagement and type	Yes	39.13 (13.948)	1.672	0.095
	No	37.44 (14.988)		
School Year	First year of specialization	38.61 (13.173)	0.873	0.558
	Second year of specialization	55.5 (11.818)		
	Third year of specialization	36.69 (15.091)		
	1st grade	38.81 (14.879)		
	2nd grade	38.52 (13.95)		
	3rd grade	39.96 (14.51)		
	4th grade	42.36 (16.041)		
	First year graduate students	34 (11.136)		
	Second year graduate students	41.33 (15.945)		
	Third year graduate students	30.67 (2.082)		
	Others	40.08 (15.723)		
Academic qualifications	College	38.58 (13.266)	0.314	0.815
	Undergraduate	38.95(14.704)		
	Master or above	39(14.104)		
	Others	41.89(19.627)		

## Discussion

The unfavorable public perception of nursing in China is influenced by cultural and educational factors. Traditionally, caregiving or service roles within Chinese culture were often designated to individuals of perceived lower social status [45]. In ancient China, a strong emphasis on gender roles led to men being expected to engage in activities outside the home, while women were tasked with domestic responsibilities. As a result, nursing, viewed as an extension of domestic care, was considered an exclusively female profession. This contributed to the perception that men working in nursing either lacked masculinity or had limited career prospects [46]. Until now, there has been a gap in research exploring nursing students’ misconceptions about male nurses.

This study marks the first attempt to address gender prejudices regarding male nurses among nursing students. Through thorough cultural adaptation and translation into Chinese, the scale’s validity was confirmed using benchmarks such as internal consistency, retest reliability, content reliability, structural and construct validity, and discriminant validity. The final product was a 17-item scale with a bifactorial structure in Chinese.

Results showed that Chinese version items matched original items, with no items being dropped. However, the factor structure of the Chinese version differs from that of the original. While the original GEMINI demonstrated a unidimensional construct in factor analyses conducted with nursing student populations, the Chinese version revealed two dimensions. The first factor, titled “Personal Development,” included 11 items from the original scales 3–6 and 11–17. The second factor, “Social Acceptance,” comprised 6 items from the original scales 1–2 and 7–10. This bifactorial structure can be attributed to several logical justifications. Firstly, the original structure was affected by cross-cultural adaptations made during the translation process, which may have altered how certain items corresponded to Chinese modes of expression. Secondly, differences in misconceptions about professional gender roles between domestic and international contexts may stem from variations in socio-cultural backgrounds and sample populations. Finally, the factor of “Social Acceptance” is influenced by traditional attitudes, misinformation in mass media, or biased perceptions of the profession, often leading to gender bias in nursing. These biases and misconceptions about men in nursing frequently affect men’s professional and career choices [1, 11]. According to social identity theory [47–50], individuals form preferences for their own group through social categorization, and prejudices against other groups. Achieving or sustaining a positive social identity improves self-esteem, which arises from favorable comparisons between the relevant in-group and out-group. Thus, we named the first dimension “Personal Development,” referring primarily to in-group preferences, and the second dimension “Social Acceptance.” [51]. The two factors of the GEMINI explained 67.883% of the variance. Except for item 1, every item had a factor loading of at least 0.50, which is considered ideal [50]. The study found that the translated scales were easily understood and well-structured, indicating that the scale’s two-dimensionality was more suitable for the Chinese population.

To assess the internal consistency and temporal stability of the scale, this study utilized Cronbach’s alpha and retest reliability. The Cronbach’s alpha of the present study exceeded that of the original version, which may reflect differences in the learning environment and cultural background of the Chinese nursing students. These results indicate strong internal correlations and commendable homogeneity among the 17 items. Additionally, the scale demonstrated high stability and reproducibility when administered to nursing students, suggesting that the Chinese version of the GEMINI is a reliable tool for assessing misconceptions about male nurses among nursing students.

The results indicated that scores on the GEMINI were higher among men and among those for whom the nursing program was not their first choice. Male nursing students in China often face misunderstandings and negative experiences from their student days through to their clinical placements, consistent with studies from other countries [52, 53]. Those who completed their obstetrics and gynecology placements were more likely to be rejected by patients and even isolated in the clinical setting [54]. Additionally, the results showed differences based on whether the nursing program was the first choice and not based on whether nursing was the first choice. This may be due to the fact that the participants in this study are nursing students, who are primarily focused on attending classes. By taking nursing courses, gender bias can be effectively reduced. Many students in lower grades or just entering university may not be aware of nursing as a profession, hence no differences were observed based on whether nursing was their first choice.

In our analysis, there was a direct correlation between the comprehensive GEMINI score and the facets of its Chinese version. Conversely, an inverse relationship was observed between the dimension scores and both the total and PIQNS scores. This suggests that a higher score on the Chinese GEMINI version is associated with a reduced PIQNS score, indicating a reduced identification with the nursing profession among nursing students. Professional identity in the context of nursing is characterized as a favorable personal disposition towards the profession, encompassing a comprehensive range of skills and responsibilities acquired during professional training [55].Furthermore, a nurse’s professional identity impacts their overall well-being and their decision to practice clinically, exacerbating the already critical issue of nursing workforce shortages.

According to the findings, the Chinese translation of the GEMINI demonstrates high levels of homogeneity, stability, structural validity, content validity, and discriminant validity. Consequently, the Chinese version of the GEMINI is suitable for evaluating misconceptions about men in nursing among Chinese nursing students.

### Limitations

This study has certain limitations. It is restricted to nursing students residing in Liaoning Province and therefore cannot be generalized to all nursing students across China. Additionally, the GEMINI scale was utilized specifically to measure gender misconceptions among male nursing students, and its applicability on a national level requires further investigation.

## Conclusions

In this study, the Chinese version of the GEMINI demonstrated good validity and reliability, featuring 17 items and a two-factor structure. It proved to be a reliable method for evaluating male nursing biases among Chinese nursing students. The GEMINI scale plays a critical role in nursing education as it identifies specific misconceptions that may affect academic performance and influence career choices for male nursing students. This insight suggests methods to help educational reform of the nursing program and society address persistent gender misconceptions, thereby improving the representation of men in the nursing field and better preparing nursing students for clinical work.

## Data Availability

The datasets generated and/or analysed during the current study are not publicly available due Chinese people are relatively secretive about their lives and thoughts, although informed consent was obtained from study subjects prior to the survey and the findings were largely reported but are available from the corresponding author on reasonable request.”

## Abbreviations

GEMINI: The gender misconceptions of men in nursing
PCA: Principal components analysis
EFA: Exploratory Factor Analysis
CFA: Confirmatory Factor Analysis
CFI: Comparative Fit Index
TLI: Tucker-Lewis Index
RMSEA: Root Mean Square Error of Approximation
GFI: goodness-of-fit index
IFI: incremental fit index
